# Trihelix transcription factor *GT*-*4* mediates salt tolerance via interaction with *TEM2* in *Arabidopsis*

**DOI:** 10.1186/s12870-014-0339-7

**Published:** 2014-12-03

**Authors:** Xiao-Hong Wang, Qing-Tian Li, Hao-Wei Chen, Wan-Ke Zhang, Biao Ma, Shou-Yi Chen, Jin-Song Zhang

**Affiliations:** State Key Lab of Plant Genomics, Institute of Genetics and Developmental Biology, Chinese Academy of Sciences, Beijing, 100101 China

**Keywords:** Salt stress, Trihelix transcription factor, GT-4, TEM2

## Abstract

**Background:**

Trihelix transcription factor family is plant-specific and plays important roles in developmental processes. However, their function in abiotic stress response is largely unclear.

**Results:**

We studied one member *GT*-*4* from *Arabidopsis* in relation to salt stress response. *GT*-*4* expression is induced by salt stress and GT-4 protein is localized in nucleus and cytoplasm. GT-4 acts as a transcriptional activator and its C-terminal end is the activation domain. The protein can bind to the *cis*-elements GT-3 box, GT-3b box and MRE4. GT-4 confers enhanced salt tolerance in Arabidopsis likely through direct binding to the promoter and activation of *Cor15A*, in addition to possible regulation of other relevant genes. The *gt*-*4* mutant shows salt sensitivity. TEM2, a member of AP2/ERF family was identified to interact with GT-4 in yeast two-hybrid, BiFC and Co-IP assays. Loss-of-function of *TEM2* exerts no significant difference on salt tolerance or *Cor15A* expression in Arabidopsis. However, double mutant *gt*-*4/tem2* shows greater sensitivity to salt stress and lower transcript level of *Cor15A* than *gt-4* single mutant. GT-4 plus TEM2 can synergistically increase the promoter activity of *Cor15A*.

**Conclusions:**

GT-4 interacts with TEM2 and then co-regulates the salt responsive gene *Cor15A* to improve salt stress tolerance.

**Electronic supplementary material:**

The online version of this article (doi:10.1186/s12870-014-0339-7) contains supplementary material, which is available to authorized users.

## Background

Plant growth, development and productivity are greatly affected by adverse environmental conditions such as drought, cold and high salinity. A plenty of genes have been reported to respond to these abiotic stresses. Among them, transcription factor genes are important for adaptation to these stresses. Several classes of transcription factors have been found to play important roles in plant stress tolerance through binding of *cis*-acting elements in the promoter region of stress-responsive genes [1-9].

The trihelix transcription factor family is defined according to the highly conserved trihelix domain which specifically binds to the GT-elements [10,11]. The trihelix domain has similarities to the individual repeats of the MYB family from which the trihelix may have been derived [12]. Compared with other transcription factors, e.g. MYB, AP2/EREBP, bHLH, NAC and WRKY families with more than 100 members in Arabidopsis, trihelix family is relatively small [13]. Until now, there are 30 members in Arabidopsis and 31 members in rice, and the members in Arabidopsis can be grouped into five classes, namely GT-1, GT-2, SH4, GTγ and SIP1. Each class is named after the relevant founding member [14,15].

Since members of trihelix family specifically bind with GT elements, these proteins are also named as GT factor. The first trihelix transcription factor was identified in pea (*Pisum sativum*) and named GT-1 factor. It binds specifically to a light-responsive GT element, named Box II/GT1box (5’-GTGT**GGTTAA**TATG-3’), in the pea *rbcS-3A* gene promoter. The core sequence 5’-GGTTAA-3’ is sufficient for light induction [16,17]. Later, GT-elements were found in many promoters of genes, some of which were not responsive for light [11]. For instance, a GT element named Site1, found in the ribosomal protein gene *rps1* promoter, represses transcription in non-photosynthetic tissues or cells [18,19]. Box II-related/GT-1 like element in the promoter region of *Pr-1A* gene from tobacco is likely responsive to pathogen infection [20]. Soybean *SCaM-4* gene with GT-1 element in the promoter region interacts with GT1-like factor and can be induced by pathogen attack and NaCl treatment [21].

Current information suggests that trihelix transcription factors not only regulate light-responsive genes [17,22-24] but also play important roles in the regulation of developmental processes involving flowers, trichomes, stomata, embryos and seeds [14,25-28] and in responses to biotic and abiotic stresses [21,29-33].

Arabidopsis *PETAL LOSS* (*PTL*), which belongs to the GT-2 group, was the first trihelix gene identified associating with a morphogenetic function. PTL regulates petal and sepal growth, and sepal fusion [25,28,34]. Rice Shattering 1(*SHA1*) gene encoding a SH4-type factor plays an important role in activation of cell separation, and a mutation in the trihelix domain results in the elimination of seed shattering in cultivated rice [26]. More recently, ASIL1, belonging to a new subfamily of trihelix family, has been found to function as a negative regulator of a large subset of Arabidopsis embryonic and seed maturation genes in Arabidopsis seedlings [14]. The role for GT-3b in responding to salt and pathogen stress is also identified in Arabidopsis. *GT-3b* expression is rapidly induced by NaCl and Pseudo-monas syringae infection and GT-3b binds to a GT-like element (GAAAAA) in the promoter of a calmodulin gene (*SCaM-4*) [21]. We demonstrates that overexpression of *GmGT-2A* or *GmGT-2B* from soybean enhanced tolerance to salt, drought and freezing stresses in transgenic Arabidopsis plants [29].

Although the roles of the GT factors are gradually disclosed, the regulatory functions of this kind of transcription factors in abiotic stress response remains largely unknown. In the present study, we find that expression of *GT-4* is induced by high salinity. Mutation of the gene causes sensitivity to salt stress and transgenic plants overexpressing *GT-4* exhibits salt tolerance compared to Col-0. We further identified a B3 and AP2/ERF domain-containing protein (TEM2) that interacted with GT-4. Loss function of *TEM2* in *gt-4* mutant affected plant performance under salt stress. The downstream gene *Cor15A* was co-regulated by GT-4 and TEM2. The GT-4 may associate with TEM2 to co-activate *Cor15A* for salt stress tolerance.

## Results

### *GT-4* expression and protein subcellular localization

**Figure 1:**
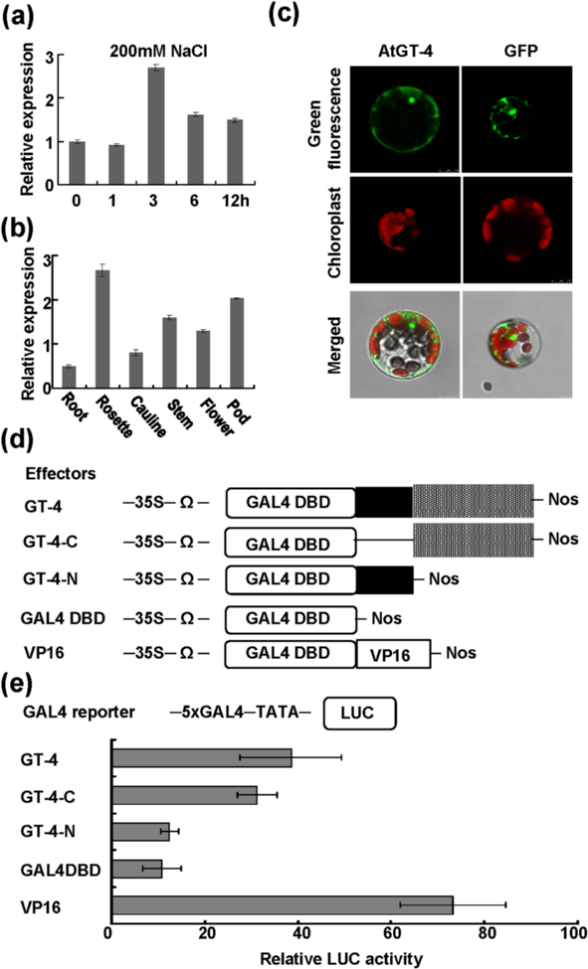
***GT-4*** **gene expression and protein localization and transcriptional activation ability. (a)** *GT-4* expression levels in response to salt stress. Bars indicate SD (n = 3). **(b)** *GT-4* expression in various organs of Arabidopsis plants. Bars indicate SD (n = 3). **(c)** Subcellular localization of GT-4 protein in Arabidopsis protoplasts. **(d)** Effector constructs used in the Arabidopsis protoplast transient assay. Each effector contained a GAL4 DNA-binding domain (GAL4DBD). The GAL4DBD effector was used as a negative control, and effector VP16, was used as a positive control. Full length GT-4, GT-4-N (1–113) and GT-4-C (114–372) was fused with the GAL4DBD and expression was driven by the 35S promoter plus the translation enhancer Ω sequence. **(e)** Transcriptional activation ability of GT-4, GT-4-N and GT-4-C as revealed by relative LUC activity of the reporter. The effectors and the GAL4-LUC reporter were co-transfected. Bars indicate SD (n = 4).

There are 26 members of GT family in Arabidopsis and we examined expressions of all of these genes in response to various stresses [35]. One of the stress-responsive genes, named *GT-4* (At3g25990), was further analyzed. *GT-4* encoded a protein of 372 amino acids and the protein had a trihelix DNA binding domain in the N-terminus and a variable C-terminus. Arabidopsis seedlings were treated with salt stress and the expression of *GT-4* was clearly induced by high salinity (Figure 1a). The expression of *GT-4* was also examined in different organs of Arabidopsis plant. Figure 1b showed that *GT-4* expressed ubiquitously and was abundant in rosette leaves and pods but less expressed in roots (Figure 1b).

We determined the subcellular localization of GT-4. *GT-4* was fused to the *GFP* gene in a transient expression vector. The fusion gene and *GFP* control driven by CaMV 35S promoter were transformed into Arabidopsis protoplasts to observe the localization of GT-4. The green fluorescence from GT-4-GFP fusion protein was localized in both nuclear and cytoplasm regions (Figure 1c). The GFP control protein was similarly localized.

### Transcriptional activation ability of GT-4

GT factors usually function as transcription factors and we measured the transcriptional activation ability of GT-4 by using a dual-luciferase reporter (DLR) assay system in Arabidopsis protoplasts [1]. Different regions of GT-4 were also examined for the activation. The domains of GT-4 were analyzed using SMART program and GT-4 protein was divided into N-terminal (amino acid No. 1 to 113, 1/113) and C-terminal (amino acid No. 114 to 372,114/372). The full length, N-terminal (1/113) and C-terminal (114/372) coding regions of GT-4 protein were fused to the GAL4 DNA-binding domain to generate pBD-GT-4, pBD-GT-4-N and pBD-GT-4-C effector plasmids respectively (Figure 1d). The fusion genes were driven by the 35S promoter plus a translational enhancer Ω. The firefly luciferase gene (*LUC*) driven by a mini-35S (TATA box) promoter with five copies of the GAL4 binding element was used as a reporter (Figure 1d), and the Renilla luciferase gene driven by the Arabidopsis *Ubiquitin3* promoter was used as an internal control. VP16, a herpes simplex virus (HSV)-encoded transcriptional activator protein was used as a positive control. The GAL4 DNA-binding domain in the fusion proteins binds to the GAL4 binding element upstream of the reporter *LUC* gene, and the activation domain in the tested proteins activates *LUC* gene transcription. Compared with the GAL4-DBD negative control, full length GT-4 and C-terminal region (114/373) of GT-4 could activate the reporter gene, whereas the N-terminal region (1/113) of GT-4 didn’t have the ability to activate reporter gene expression (Figure 1e). The results indicated that GT-4 and its C-terminal domain possess transcriptional activation ability.

### DNA-binding ability of GT-4

**Figure 2:**
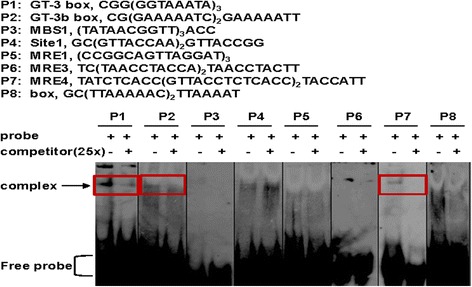
**DNA binding ability of GT-4.** Upper panel: various elements used for GT-4 protein binding assay. Lower panel: GT-4 was expressed and subjected to a gel-shift assay. GT-4 can bind to P1, P2 and P7 elements. The arrowhead indicates the positions of a protein/DNA complex.

GT proteins specifically bind to GT elements, and the elements are highly degenerated. GT-1 and GT-3 proteins with one trihelix DNA-binding domain specially bind to Box II core sequence (5’-GTGTGGTTAATATG-3’) and the 5’-GTTAC-3’ sequence respectively. GT-2 protein with two trihelix DNA-binding domains can bind to GT-2 box (5’-GCGGTAATTAA-3’) and GT-3 box (5-GAGGTAAATCCGCGA-3) sequences [17,36,37]. There are reports that trihelix proteins resemble those of MYB proteins [12]. Several known GT elements and MYB protein binding elements were selected as binding elements (P1 to P8) to identify the DNA-binding ability of the present GT-4 by EMSA (Figure 2, upper panel). GT-4 formed a complex with P1 (GT-3 box), P2 (GT-3b box) and P7 (MRE4), and the intensity of the retarded bands were dramatically reduced when non-labeled competitors were included (Figure 2, lower panel), indicating that GT-4 specifically binds to these elements. It should be noted that the GT-4 may also bind to the P3 (MBS1) and P8 (box) probes since addition of the competitors seemed to reduce the band intensities slightly (Figure 2).

### Performance of mutant *gt-4* and transgenic Arabidopsis plants overexpressing *GT-4* under salt stress

**Figure 3:**
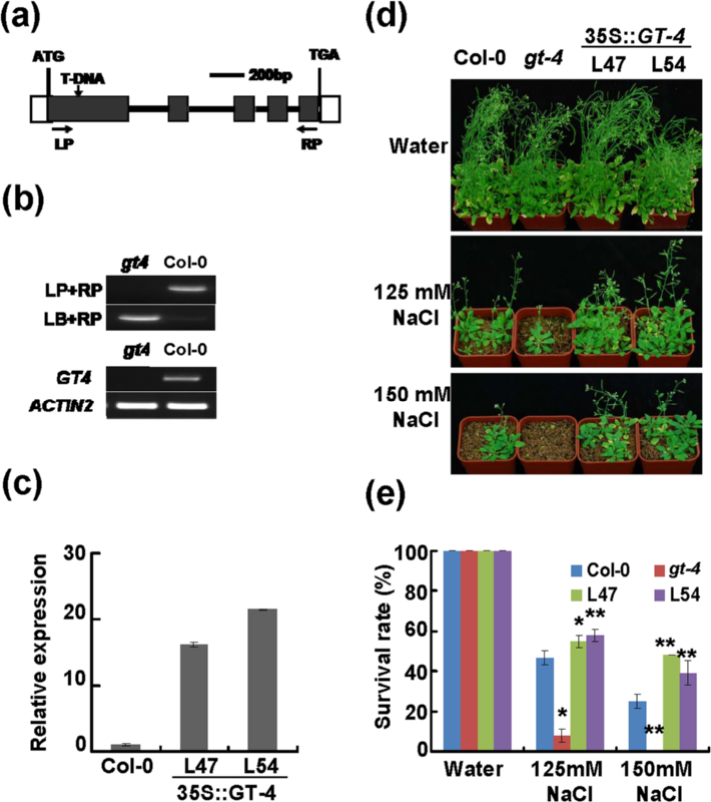
**Identification of** ***gt-4*** **mutant and** ***GT-4*** **-overexpressing lines and performance of these plants under salt stress. (a)** T-DNA insertion site in *GT-4* in the *gt-4* mutant. The filled black boxes represent ORFs, while the lines between the boxes represent introns. LP and RP are primers used for PCR analysis. **(b)** RT-PCR analysis of the *GT-4* transcript levels in seedlings of Col-0 and mutant lines. The *Actin2* gene was used as an internal control. **(c)** *GT-4* transcripts in Col-0 and *GT-4*-over-expression plants by qRT-PCR analysis. Bars indicate SD (n = 3). **(d)** Performance of various plants under salt stress. Seven-day-old plants were transferred to NaCl-containing pot and grew for 5 weeks. **(e)** Survival rates of plants after salt treatments. Bars indicate SD (n = 4). Each replicate uses 16 plants. Asterisks indicate a significant difference compared to the corresponding Col-0 (*P <0.05 and **P <0.01).

To elucidate the biological function of *GT-4*, one T-DNA insertion mutant was identified and designated as *gt-4* (SALK_095404). The T-DNA insertion was located in the first exon of *GT-4* (Figure 3a) and was confirmed by PCR (Figure 3b). No full-length transcript of *GT-4* was detected in the mutant *gt-4* by RT-PCR (Figure 3b), suggesting that *gt-4* was loss-of-function mutant. Transgenic Arabidopsis plants overexpressing *GT-4* driven by the CaMV 35S promoter were generated. At least 60 transgenic lines were obtained, and two independent homozygous lines (*GT-4*-OE) L47 and L54 with relatively high expression of *GT-4* (Figure 3c) were further investigated. Since *GT-4* was responsive to salt stress, we tested if it is involved in regulation of stress tolerance. Under normal growth condition, mutant *gt-4*, *GT-4*-OE and Col-0 plants showed normal growth (Figure 3d). All plants (7-day old) were transferred to soils in pots saturated with NaCl solutions and grew for 5 weeks (Figure 3d). The *gt-4* mutants were more sensitive to salt and transgenic plants were more tolerant to salinity than Col-0 as can be seen from both the growth performance and the survival rate under 125 mM and 150 mM NaCl treatments (Figure 3d,e). These results indicate that *GT-4* plays a positive role in the regulation of plant tolerance to salt stress.

### GT-4 regulates expressions of *Cor15A*

**Figure 4:**
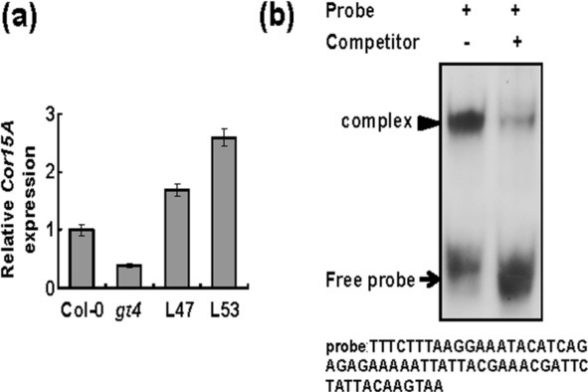
***GT-4*** **regulates stress-responsive gene** ***Cor15A*** **. (a)** *GT-4* regulates *Cor15A* expression as revealed by quantitative PCR. Each column is the mean of four replicates. L47 and L53 indicate transgenic lines overexpressing *GT-4*. 9-day-old plate-grown seedlings of Col-0, *gt-4*, and transgenic lines overexpressing *GT-4* were used for RNA extraction and reverse-transcription. Bars indicate SD (n = 3). **(b)** GT-4 specifically binds to the *Cor15A* promoter region. The arrowheads indicate the position of the protein/DNA complex.

Expression of stress-related genes was examined in mutant *gt-4* and *GT-4-* overexpressing plants grown under normal condition by quantitative PCR. Compared with that in Col-0, the expressions of *Cor15A* (At2g42540) was enhanced in *GT-4*-OE lines but decreased in *gt-4* plants (Figure 4a). The result implies that GT-4 may confer stress tolerance through activation of *Cor15A*. We determined whether GT-4 regulates *Cor15A* by direct binding to its promoter region and the EMSA was performed. Since GT protein can bind to GT-3b box, GT-4 may bind to the same element in the promoter region of downstream genes. A 60 bp DNA fragment from the *Cor15A* promoter was identified to contain the GT-3b box. GT-4 was found to specifically bind to this sequence from *Cor15A* promoter (Figure 4b). These results indicate that GT-4 most likely activates *Cor15A* expression through direct binding to the GT-3b box in its promoter region.

### GT-4 interacts with TEM2, a protein with B3 and AP2/ERF domain

**Figure 5:**
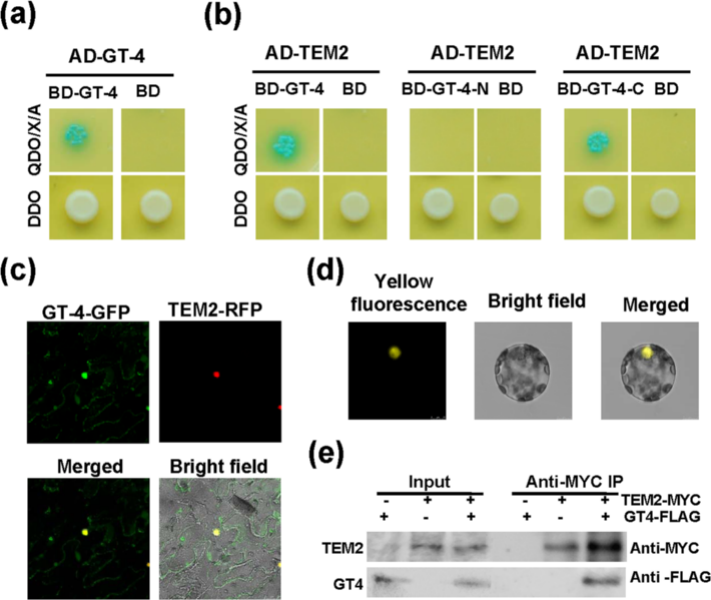
**GT-4 interacts with TEM2 in vivo and in vitro. (a)** Dimerization of Arabidopsis GT-4 in yeast two-hybrid assay. The yeast cells harboring the plasmid combinations were grown on DDO plates (SD medium without Leu and Trp) and QDO/X/A plates (SD/–Ade/–His/–Leu/–Trp medium containing 40 μg mL-1X-α-Gal and 125 ng mL-1 Aureobasidin A) for 3 d. The cells generating blue color indicate positive interactions between the two proteins. **(b)** GT-4 and C-terminal of GT-4 interact with TEM2 in yeast two hybrid assay. **(c)** GT-4 and TEM2 co-localized in the nucleus. Tobacco leaves were co-infiltrated with 35S:GFP-GT-4 and 35S:RFP-TEM2. Signals were observed directly under a confocal microscope after 3 days. **(d)** Bimolecular fluorescence complementation (BiFC) assay. The fusion constructs were co-transformed into Arabidopsis protoplasts and the cells were observed 16 h later under a confocal microscope. YFP fluorescence was excited at a wavelength of 488 nm. **(e)** Co-IP assay. Nuclei were isolated from tobacco leaves expressing 35S:GT-4-FLAG and 35S:TEM2-MYC. Then proteins were extracted and incubated with anti-c-Myc agarose beads. The proteins were then eluted and followed by western blotting analysis with anti-flag or anti-Myc antibodies.

Transcription factors were reported to interact with the same family proteins or other transcription factors [38]. The proteins interacted with GT-4 were identified by using a yeast two-hybrid assay system. GT-4-coding sequence was cloned into pGBKT7 vector and the recombinant BD-GT-4 protein was used as a bait to screen an Arabidopsis prey cDNA library. Among four unique genes encoding putative GT-4-interacting proteins, a cDNA encoding a transcription factor TEM2 (At1g68840) containing an AP2/ERF domain was selected for further investigation. To clarify the interaction, the coding sequence of *TEM2* was fused to the 3’-end of the GAL4 activation domain (AD) coding region to generate pGADT7-TEM2. Combinations corresponding to AD-TEM2 mating with BD-GT-4 showed a clear positive interaction on the QDO/X/A plate (Figure 5b). We also investigated the interacting domain of GT-4 with TEM2, and found that the C-terminal but not the N-terminal end of GT-4 interacted with TEM2 (Figure 5b). Furthermore, GT-4 can form homo-dimer itself (Figure 5a). BD-GT-4, BD-GT-4 N, BD-GT-4C, AD-GT-4 and AD-TEM2 fusion plasmids were respectively transfected into the yeast cells as negative controls and the corresponding proteins showed no auto-activation or DNA-binding abilities (see Additional file 1).

To visualize the co-localization of GT-4 and TEM2, the full-length coding sequences (CDS) of *GT-4* and *TEM2* without stop codon were cloned into pGWB405 and pGWB454 vectors with C-terminal sGFP and mRFP fusion respectively. Confocal analysis revealed that the GT-4 and TEM2 co-localized in the nucleus (Figure 5c).

A bimolecular fluorescence complementation (BiFC) system was used to further characterize the interaction between GT-4 and TEM2 in *planta*. GT-4 tagged with C-terminal (YC) and TEM2 tagged with N-terminal of yellow fluorescent protein (YN) were transiently co-expressed in Arabidopsis protoplasts, and the yellow fluorescence was detected in the nuclei of Arabidopsis protoplasts (Figure 5d). However, YC-GT-4 and YN or YN-TEM2 and YC did not exhibit any fluorescence (see Additional file 2). These results suggest that GT-4 can interact with TEM2 in the nuclei of plant cells.

To further confirm the interaction between GT-4 and TEM2 in vivo, a Co-immunoprecipitation (Co-IP) assay was performed. Two constructs 35S-Myc-TEM2 and 35S-GT-4-Flag were produced using the Gateway system. Argo-infiltration procedure was then performed with minor modifications. Proteins were isolated from tobacco leaves expressing 35S-Myc-TEM2 and 35S-GT-4-Flag, and then incubated with anti-c-Myc agarose beads. The proteins were then eluted and followed by western blotting analysis with anti-Flag or anti-Myc antibodies. Figure 5e showed that GT-4 interacted with TEM2 in an in vivo assay.

### The GT-4 and TEM2 affect salt stress response via co-regulation of *Cor15A*

**Figure 6:**
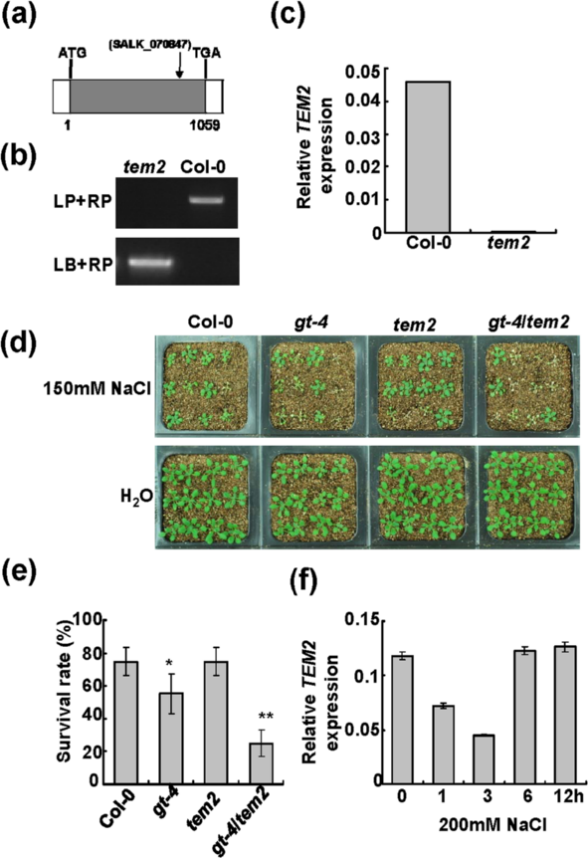
**Performance of various single and double mutants in response to salt stress. (a)** The T-DNA insertion site in *TEM2* gene of the *tem2* mutant. The filled grey box represents the ORF. **(b)** T-DNA insertion confirmation of the *tem2* mutant. **(c)** *TEM2* transcripts in Col-0 and mutant plants by qRT-PCR. The *Actin2* was used as an internal control. Bars indicate SD (n = 3). **(d)** Performance of Col-0, *gt-4*, *tem2* and *gt-4*/*tem2* plants under salt stress. 9-day-old seedlings were transferred to soil saturated with water or 150 mM NaCl and grew for 10 days. **(e)** Survival rates of plants after salt treatments. Bars indicate SD (n = 4). Asterisks indicate a significant difference compared to Col-0 (*P <0.05 and **P <0.01). **(f)** *TEM2* expression levels in response to salt stress. Bars indicate SD (n = 3).

**Figure 7:**
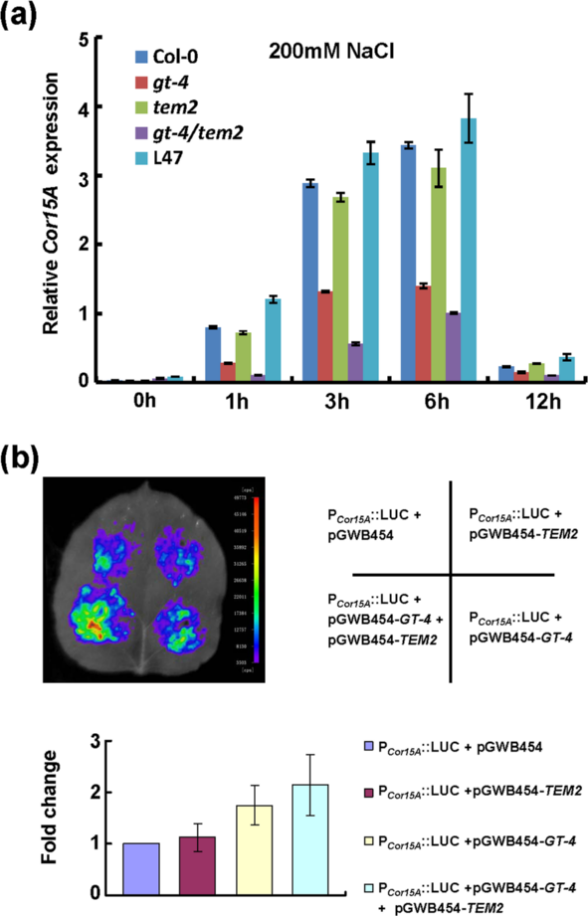
**Interaction of GT-4 and TEM2 activates** ***Cor15A*** **expressions. (a)** *Cor15A* transcripts in Col-0, mutant plants and L47 with 200 mM NaCl treatment for 1 h, 3 h, 6 h and 12 h. Bars indicate SD (n = 3). **(b)** GT-4 plus TEM2 activate *Cor15A* promoter activity in tobacco leaves. The *A. tumefacien* harboring *P*_*Cor15A*_::LUC were mixed with *A. tumefacien* harboring pGWB454, pGWB454-*GT-4* and pGWB454-*TEM2* respectively and infiltrated into tobacco leaves. LUC image was taken 2 days after infiltration. Fluorescence intensity was analyzed with IndiGo software (lower panel). Each column is the mean of more than 5 leaves. Bars indicate SD.

Previous reports have found that TEM2 acts as a transcriptional repressor and is regulated by light and the circadian clock. Over-expression of *TEM2* severely delayed the flowering time of Arabidopsis [39], yet the role of *TEM2* in salt stress response is unclear. We thus analyzed the function of TEM2 in salt stress responses. The expression of *TEM2* was suppressed after initiation of salinity and then recovered to normal level subsequently (Figure 6f). One T-DNA insertion mutant was identified and designated as *tem2* (SALK_070847). The insertion was located in the only exon (Figure 7a) and was confirmed by PCR (Figure 6b). No *TEM2* expression was detected in the mutant by qRT-PCR (Figure 6c), suggesting that *tem2* is a loss-of-function mutant.

To further analyze the mechanism of GT-4 and TEM2 in salt stress response, *gt-4* mutant was crossed with *tem2* mutant to obtain double mutant *gt-4/tem2*. We then examined the salt stress response of Col-0, *gt-4*, *tem2* and *gt-4/tem2*. The 10-day-old Col-0 and mutant plants were transferred into soil saturated with 150 mM NaCl and maintained for a period of 10 d. No obvious difference was observed between Col-0 and *tem2*, whereas a remarkably low number of *gt-4/tem2* plants were found to survive the exposure to salinity in comparison with the control and *gt-4* plants (Figure 6d,e). Under normal condition, no obvious difference was observed between Col-0 and various mutants (Figures 6d).

Real-time quantitative PCR analysis was performed to examine the expressions of *Cor15A* in Col-0, *gt-4*, *tem2*, *gt-4/tem2* mutant plants and *GT-4*-OE line 47 (L47) under salt stress. The Col-0 and *tem2* displayed similar levels of *Cor15A* expression under salt stress. The *gl-4* mutant showed an approximate two-fold decrease of *Cor15A* expression compared to the Col-0 level throughout the treatments. In the double mutant *gt-4/tem2*, more severe decrease of *Cor15A* expression was observed than those in the other plants tested. *Cor15A* transcript level in L47 was always higher than that in Col-0 (Figures 7a).

We further confirmed qPCR results by an in vivo transient luciferase expression system. The *Cor15A* promoter was fused to the LUC reporter, and the resulting construct was co-transformed into tobacco leaves with pGWB454-TEM2 and/or pGWB454-GT-4. We found that GT-4 could activate the *Cor15A* promoter activity whereas TEM2 didn’t significantly affect this activity. The activation was stronger when these two proteins were co-expressed (Figures 7b). These results indicate that GT-4/TEM2 complex are transcriptional activator of *Cor15A* expression to regulate salt response.

## Discussion

Previously, we have found that soybean trihelix transcription factors *GmGT-2A* and *GmGT-2B* improve plant tolerance to abiotic stresses in transgenic Arabidopsis [29]. *GT2L* was reported to interact with calmodulin and respond to salt stress and regulate expression of *RD29A* and *ERD10* [40]. In the present study, *GT-4* was identified to be stress-responsive and conferred salt tolerance in transgenic Arabidopsis plants through regulation of downstream gene *Cor15A*. The *gt-4* mutant was sensitive to salt stress as revealed by phenotypic change. GT-4 binds to the GT-3b box in the *Cor15A* promoter and activates its expressions. A B3 and AP2/ERF domain containing protein TEM2 was identified to interact with GT-4 and jointly regulate salt tolerance.

Various binding elements of trihelix proteins have been identified. Previous study showed that the GT-4 recombinant protein binds to the GT1-box, GT2-box and GT3-box [15,41]. However, the positions of the indicated GT-4 protein/DNA complex are very low and near to the free probe regions [41]. The *GT-4* gene is also induced by light [41]. In our present study, we found that GT-4 not only binds to the GT-elements, such as GT-3 box and GT-3b box, but also bind to the MYB protein binding element MRE4 with weaker affinity (Figure 2). In addition, the position of our protein/DNA complex is high compared to that from Murata et al. [41]. This discrepancy is likely due to the fact that we used probe elements with two or three repeats whereas Murata et al. (2002) used only one repeat [41]. Moreover, the higher positions of the protein/DNA complex in Figure 3 may also be due to the dimerization of the GT-4 protein since it has the ability to form dimers (Figure 6a).

Transcription factors have either transcriptional activation or suppression activities. Hao et al. (2011) reported that GmNAC11 had transcriptional activation activity whereas GmNAC20 had suppression activity, and have identified a NARD domain in plant NAC-type transcription factors for suppression of transcriptional activation [1]. Cotton TCP protein GhTCP14 contains transcriptional activation activity, however, it can directly down-regulate the expression of *PIN2* possibly by its interaction with other proteins [42]. A rice GT-2 protein has been found to function as a transcriptional activator. However, the activation domain was not identified [43]. Arabidopsis GT-1 also has transactivation function in both yeast and plant cells [44]. GTL1 functions as a transrepressor for *SDD1* gene in stomatal development in Arabidopsis [31]. Presently, we find that GT-4 exhibited transcriptional activation activity in protoplast assay and the activation domain may be located in the C-terminal end (amino acids 114–372) of GT-4 (Figure 1b). The N-terminal end (amino acids 1–113) containing the trihelix domain may function as the DNA-binding domain. The minimal domain for transcriptional activation needs further study.

GT-4 may enhance stress tolerance by regulating downstream stress-responsive gene *Cor15A* through direct binding to the GT-3b box in the *Cor15A* promoter (Figure 4). The Cor15A protein shows homology to the LEA protein family. The Cor15A may protect lactate dehydrogenase from aggregation during stress [45,46]. Overexpression of the *Cor15A* gene in Arabidopsis leads to accumulation of the protein in chloroplast stroma and confers freezing tolerance [47]. Transgenic Arabidopsis overexpressing *Cor15A* exhibited greater NaCl tolerance than the wild-type in saline soil [48]. Therefore, increased expression of *Cor15A* in *GT-4* overexpression plants may provide protection by preventing damage of chloroplast membrane and enzymes from salt stress. It should be noted that in *GT-4* overexpressing plants, the *GT-4* expression is enhanced by around 15-fold while the *Cor15A* level is only enhanced by 2-fold (Figure 4). This discrepancy may be due to that the GT-4 protein levels and/or its activities are not elevated in proportion. Alternatively, other relevant downstream genes may also be regulated for stress responses.

Most trihelix proteins have been identified to localize to the nucleus [25,29,37,49,50]. GT-4 has been reported to localize to the nucleus of the onion epidermal cells [41]. However, in the present study, it should be mentioned that GT-4 not only localize in the nucleus but also in the cytoplasm (Figures 1e, 5c), suggesting that GT-4 may have other regulatory roles in the cytoplasm in addition to its function as a transcriptional activator in the nucleus and it needs to be further studied.

Through yeast two-hybrid assay, BiFC and Co-IP, we find that GT-4 could interact with TEM2 and the interaction seems to be mediated by the C-terminal of GT-4 (Figure 5a and b). TEM2 may enhance the activation activity of GT-4 via interacting with C-terminus activation domain (Figure 5b, 7b). It has been reported that Arabidopsis GT-3a and GT-3b could form homo or heterodimers, and the dimerization domain seemed to be located at the C-terminus [37]. Likewise, GT-4 can interact with itself that may function as homo-dimer to regulate gene expression. It is possible that GT-4 dimerization may facilitate the association of GT-4 with TEM2, and whether this is the case remains to be further studied. TEM2 is a member of RAVE subfamily of AP2-EREBP family and play an important role in flower development and mechanical stimuli response [39,51]. Members of AP2-EREBP family have been shown to be involved in enhancement of salt tolerance [52-54].

*Cor15A* expression increases in Arabidopsis in response to NaCl with transcript levels peaking at 6 h and returning to near basal levels within 12 h. The expression levels of *Cor15A* roughly correlate with the survival rates of the double and single mutants after salt treatment (Figures 6e, 7a). The above results seem to be consistent with the ability of GT-4, TEM2 or their combination in activation of *Cor15A* promoter activities (Figure 7b).

The salt-inductions of *Cor15A* expression in *tem2* mutant is largely unchanged, suggesting that there is no obvious correlation between *Cor15A* expression and *TEM2* (Figure 7a). Additionally, overexpression of *TEM2* exerts no influence on P_*Cor15A*_::LUC (Figure 7b). However, the double mutant *gt-4/tem2* show a more severe reduction in *Cor15A* expression and salt tolerance than *gt-4* single mutant (Figures 6d,e and 7a), indicating that, under high-salinity condition, activation of *Cor15A* and promotions of tolerance by elevated *GT-4* expression partially depend on *TEM2* function and TEM2 regulate salt stress response through interaction with GT-4 on the whole. It is possible that the interaction between GT-4 and TEM2 would enhance the transcriptional activation ability of GT-4 and then promote *Cor15A* expressions. This finding raises the possibility that GT-4 acts as a transcription activator in cooperation with the TEM2 under salinity condition and confers stress tolerance. It should be noted that, under normal condition, the *Cor15A* expression levels increase in the *gt-4/tem2* mutant, probably reflecting a feedback mechanism for maintainment of the normal growth and development.

## Conclusions

Collectively, we demonstrate that *GT-4* has important roles in adaptation to salt stress through regulating *Cor15A*. TEM2, as a novel GT-4-interacting partner with B3 and AP2/ERF domains, also participates in salt stress responses via interaction with GT-4. Our results reveal mechanisms of *GT-4* in salt stress tolerance and provide novel gene resources for crop improvement.

## Methods

### Plant growth and treatments

Arabidopsis seeds (Columbia ecotype, Col-0) were surface-sterilized, plated on 1/2 Murashige and Skoog (MS) medium, stratified at 4°C for 3–4 d and then germinated at 23°C under 16 h photoperiod. For salt stress, 6-day-old seedlings from Col-0, *gt-4*, *tem2* and transgenic lines overexpressing *GT-4* were transferred into soil containing various concentrations of NaCl. For each NaCl treatment, at least three replicates were performed.

### Identification of T-DNA insertion mutants

T-DNA insertion mutant of *gt-4* (SALK_095404) and *tem2* (SALK_070847) was obtained from SALK database. The seed samples were sowed on 1/2MS medium. PCR screening for insertions was carried out using gene specific primer pairs. GT-4-LP is 5’-TGAGATCAATACCTTCAACAGATG-3’ and GT-4-RP is 5’- TTGTGTGCTGTTTGTTCGAAG- 3’; TEM2-LP is 5’-GTGTTGTTCCTCAGCCTAACG-3’ and TEM2-RP is 5’- TTTCCACAAAACCATTGTTCC-3’. RT-PCR analysis of full length gene expression was used to evaluate the effect of insertion. The *Actin2* was amplified as a control.

### Generation of transgenic Arabidopsis plants

The coding region of *GT-4* was amplified from cDNA with primers containing BamHI/HindIII sites, and cloned into the pCAMBIA1302 vector. The gene was driven by the 35S promoter. The forward primer is 5’-CGGGATCCATGTTTGTTTCCGATAAC -3’ and the reverse primer is 5’-CCCAAGCTTTCTCATTCCTCTGTATAAG-3’. The expression plasmid pCAMBIA-*GT-4* was transfected into agrobacterium GV3101 and then transformed into Arabidopsis plants using floral dip method. T3 homozygous plants with higher level of transgene expression were used for further analysis.

### qRT-PCR analysis

Total RNA from aerial parts of four-week-old plate-grown plants was used for reverse-transcription (RT) with MMLV reverse transcriptase according to the manufacture’s protocol (Promega). Genes selected and corresponding primers were shown in Additional file 3. Real-time quantitative PCR was performed on Roche Light Cycler 480 using the SYBR green PCR kit (TOYOBO, Osaka, Japan) and PCR was conducted according to the following protocol: 15 s denaturation at 94°C, 15 s annealing at 57°C, 15 s elongation at 72°C in 40 cycles. Fluorescence was detected at 80°C. Samples were analyzed in triplicate using independent cDNA samples and were quantified by the comparative cycle threshold method [55].

### Subcellular localization analysis

The open reading frame (ORF) of *GT-4* was amplified by RT-PCR with the specific primers 5’-CGGGATCCATGTTTGTTTCCGATAAC-3’ and 5’-CCCAAGCTTTCTCATTCCTCTGTATAAG-3’. The PCR products were digested with BamH I and HindIII, and fused to *GFP* in pBIN221. The fusion gene and the *GFP* control were driven by the 35S promoter. The two constructs were transformed into Arabidopsis protoplasts with polyethylene glycol (PEG) treatment, and GFP signal was detected by Leica TCS SP5 microscope.

### Transcriptional activation assay in Arabidopsis protoplasts

Full length and N- and C- terminal sequences of *GT-4* were obtained by PCR. For full length sequence, the forward primer is 5’-CGGGATCCATGTTTGTTTCCGATAAC-3’ and the reverse primer is 5’-GGGGTACCCCTCTCATTCCTCTGTATAAG-3’; for N-terminal sequence, the forward primer is 5’- GCTCTAGAATGTTTGTTTCCG ATAAC-3’ and the reverse primer is 5’-GGGGTACCCTCTTTCAATA TGTTCCTC-3’; for the C-terminal sequence, the forward primer is 5’-GCTCTAGATTTAAGAAAGCTAAGCAAC-3’ and the reverse primer is 5’-GGGGTACCTCATCTCATTCCTCTGTATAAG-3’. The GAL4 DNA-binding domain (BD)-coding sequence was fused to the above genes and inserted into the pRT107 to generate effector plasmids pRT-BD-GT-4. The fusion genes were under the control of 35S promoter. The BD sequence was also fused to VP16 gene to generate positive control effector plasmid. The pRT107 containing the BD sequence was used as negative control. The reporter plasmid containing 5xUAS and 35S promoter upstream of a reporter gene encoding a firefly luciferase (LUC) was used. The effector and reporter plasmids were co-transfected into Arabidopsis protoplasts and the relative LUC activity was determined based on previous descriptions [56]. The experiments have been repeated independently for three times and the results were consistent. Results from one experiment were presented.

### EMSA assay

The GT-4 coding region was amplified and cloned into the BamHI/XhoI sites of the pGEX6p-1 vector containing a GST tag. The forward primer is 5’-CGGGATCCATGTTTGTTTCCGATAACAAC-3’ and the reverse primer is 5’- CCGCTCGAGTCTCATTCCTC TGTATAAGC-3’. The GST-GT-4 fusion protein was expressed in Escherichia coli (BL21) and purified using Glutathione Sepharose 4B (GE). Oligonucleotides and their reverse complementary oligonucleotides were synthesized, and the sequences are shown in Figure 3. Double-stranded DNA was obtained by heating oligo-nucleotides at 70°C for 5 min, and annealing at room temperature in 50 mM NaCl solution. The gel-shift assay was performed as described previously [57] using digoxigenin-labeled probes. For the experiment in Figure 4b, the Arabidopsis *Cor15A* promoter sequence was used.

### Yeast two-hybrid interaction assay

The cDNA library was constructed using a Make Your Own “Mate & Plate” Library System (Clontech, Mountain View, CA, USA) according to the manufacturer's instructions. Briefly, total RNA was extracted from 2-week-old Arabidopsis seedlings air-dried for 3 h and 200 mM NaCl treated for 6 h. Library cDNAs were integrated into the linearised pGADT7-Rec vector by co-transformation and recombination in yeast strain Y187. The coding sequence of GT-4 was amplified by PCR using gene-specific primers containing NdeI and EcoRI sites. The forward primer is 5’- GGAATTCCATATGATGTTTGTTTCCGATAAC-3’ and the reverse primer is 5’- GGAATTC TCATCTCATTCCTCTGTATAAGCG-3’, and fused to the coding sequence of DNA binding domain of GAL4 to construct plasmids pGBKT7-GT-4. A yeast strain Y2HGold transformed with pGBKT7-GT-4 was mixed with pre-transformed Y187 library and the mated strains were selected on high stringency medium (SD/–Ade/–His/–Leu/–Trp medium containing 40 μg mL-1 X-α-Gal and 125 ng mL-1 Aureobasidin A). Plasmid DNA was isolated from the positive clone and the cDNA fragment was sequenced.

### Co-localization analysis in *Nicotiana benthamiana*

The full-length coding sequence (CDS) of *GT-4* and *TEM2* without stop codon were amplified and inserted into the Gateway entry vector and then cloned into pGWB405 and pGWB454 vectors with C-terminal sGFP and mRFP fusion. The argo-infiltration was performed as described below and a Leica TCS SP5 confocal laser scanning microscope was used for confocal assay. Wavelengths to visualize GFP and RFP were 500 to 540 and 600 to 640 nm, respectively.

### Bimolecular fluorescence complementation (BiFC) assay in Arabidopsis protoplasts

The ORFs of *GT-4* and *TEM2* were amplified by PCR using gene-specific primers and fused to the 5’ end of N-terminal or C-terminal of yellow fluorescent protein (YFP) coding region to form GT-4-YFPc and TEM2-YFPn. For the BiFC protein-protein interaction analysis, the above constructs were introduced into Arabidopsis protoplasts via PEG-mediated transformation, and the transgenic protoplasts were observed with a laser scanning confocal microscope (Leica, Germany).

### Co-immunoprecipitation in *Nicotiana benthamiana*

Two constructs pGWB411-10XMyc-GT-4 and pGWB421-Flag-TEM2 were made using the Gateway system. Argo-infiltration procedure was performed according to Liu et al. [58] with minor modifications. Briefly, Agrobacterium EHA105 carrying Myc-GT-4 and flag-TEM2 plasmids were suspended in infiltration buffer to OD600 = 1.0, except that p19 was diluted to OD600 = 0.6. For co-infiltration, equal volumes of different Agrobacterium strains were mixed prior to infiltration. The infiltrated parts of leave samples were taken after 48-72 h, and then ground in liquid nitrogen. Nuclei was extracted with Nuclei isolation buffer (0.25 M sourcose, 15 mM PIPES, pH6.8, 5 mM MgCl_2_, 60 mM KCl, 15 mM NaCl, 1 mM CaCl_2_, 0.9% Triton X-100, 1 mM PMSF, 2 μg/ml Pepstatin A, 2 μg/ml Aprotinin). Fine powder of each sample were suspended with gentle rotation, then left on ice for 20 min and filtered by gauze. The colatuies were centrifuged at 11000 g at 4°C for 20 minutes. The sediments were suspended by 400 μL IP buffer (50 mM Tris–HCl, PH7.6, 150 mM NaCl, 0.5% NP-40, 0.2% Triton X-100, 2 mM EDTA, and 1× protease inhibitor cocktail [Roche]). The lysates were left on ice for 2 h with gentle rotation to make proteins solubilized and centrifuged at 16500 g at 4°C for 30 minutes. Supernatants (300 μL) were performed in accordance to the manufacturer’s instructions (Thermo Pierce) and incubated with 5 μg anti-c-Myc agarose beads for overnight at 4°C with slight shaking. To elute the proteins, 25 μL 2× non-reducing sample buffer were added and heated at 95°C for 5 minutes. The presence of the Flag-TEM2 was examined with the anti-Flag antibody (EARTHOX) by western blotting.

### Detection of luciferase activity in tobacco leaf

The assay was performed as described [57]. The 1.5-kb promoter of *Cor15A* was amplified by PCR using primers (forward: 5’- ATGAAACTGAATAAACTCCCTG-3’; reverse: 5’- GAGAGAGATCTTTAAGATGTGAGA-3’) and inserted into pGWB435 to generate promoter::LUC reporter constructs using Gateway system (Invitrogen, USA). The reporter construct (P_*Cor15A*_::LUC) and the effector constructs (pGWB454, pGWB454-*GT-4*, pGWB454-*TEM2*) were transformed into A. tumefaciens strain GV3101 and introduced into tobacco leaves by infiltration. LUC activity was observed with a cold CCD camera (Berthold Technologies, Germany) 2 d after infiltration.

### Statistical analysis

The data of survival rate in Figure 3e and Figure 6e were subjected to Student’s *t*-test analysis using SPSS 11.5 (SPSS Inc., USA).

### Availability of supporting data

The data sets supporting the results of this article are included within the article and its additional files.

## Additional files

Additional file 1:
**Auto-activity of BD-GT-4, BD-GT-4N, BD-GT-4C, AD-GT-4 and AD-TEM2 in the yeast assay.**
Additional file 2:
**Negative control of bimolecular fluorescence complementation (BiFC) assay.**
Additional file 3:
**Primers used for Q-PCR.**
