# Supplementary figures and images for: Trihelix transcription factor GT-4 mediates salt tolerance via interaction with TEM2 in Arabidopsis
Source: BMC Plant Biol. 2014 Dec 3;14:339. doi: 10.1186/s12870-014-0339-7 (PMC4267404; doi:10.1186/s12870-014-0339-7)

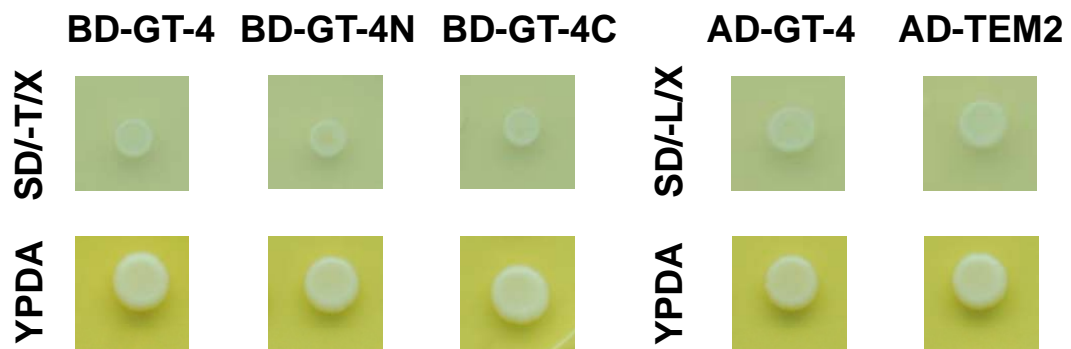

**Additional file 1.** Auto-activity of BD-GT-4, BD-GT-4N, BD-GT-4C, AD-GT-4 and AD-TEM2 in the yeast assay

Supplement: Additional file 1: — Auto-activity of BD-GT-4, BD-GT-4N, BD-GT-4C, AD-GT-4 and AD-TEM2 in the yeast assay. [file 12870_2014_339_MOESM1_ESM.pdf]

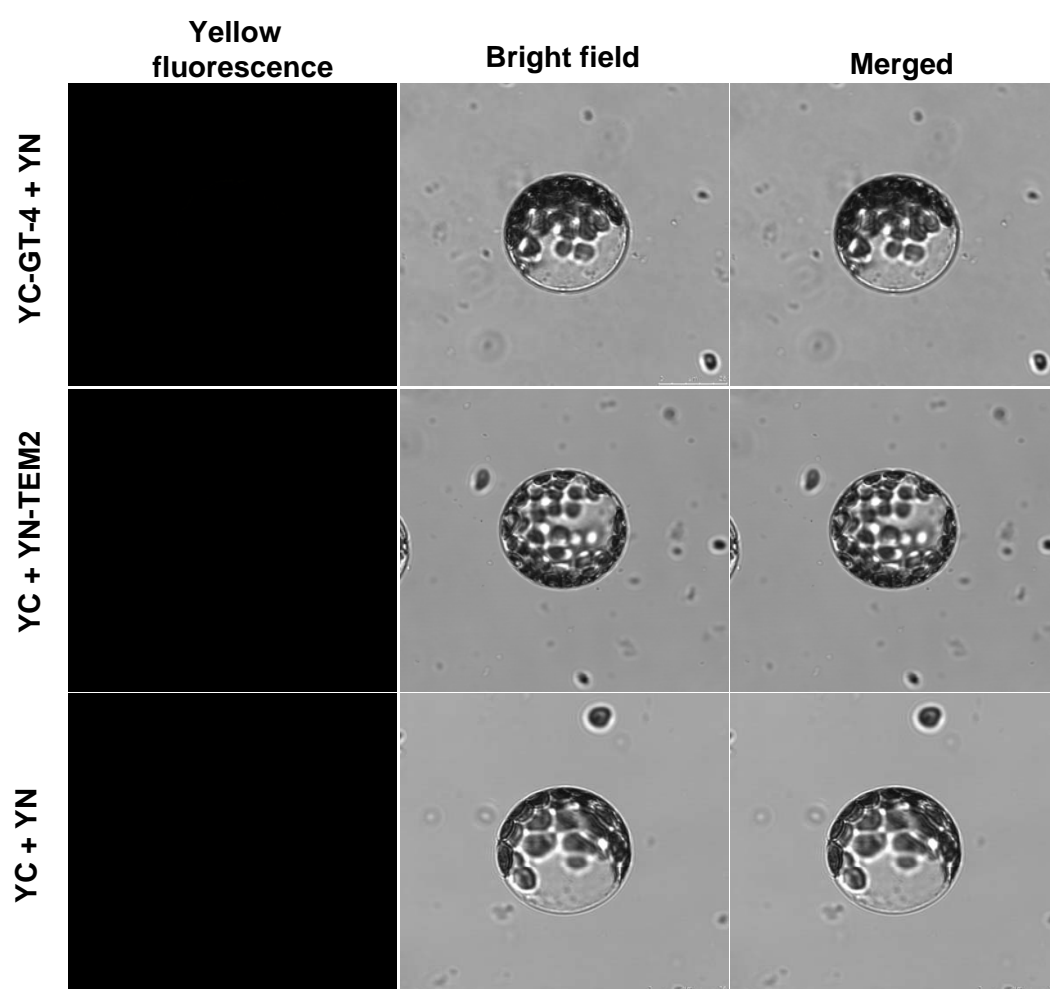

**Additional file 2.** Negative control of Bimolecular fluorescence complementation (BiFC) assay

Supplement: Additional file 2: — Negative control of bimolecular fluorescence complementation (BiFC) assay. [file 12870_2014_339_MOESM2_ESM.pdf]
