# Supplementary material for: Trihelix transcription factor GT-4 mediates salt tolerance via interaction with TEM2 in Arabidopsis
Source: BMC Plant Biol. 2014 Dec 3;14:339. doi: 10.1186/s12870-014-0339-7 (PMC4267404; doi:10.1186/s12870-014-0339-7)
Supplement: Additional file 3: — Primers used for Q-PCR. [file 12870_2014_339_MOESM3_ESM.pdf]

Additional file 3. Primers used for Q-PCR

Gene	Primer name	Primer sequence
GT-4	qGT-4 F	ACAACATCCTTCACGGGACATA
	qGT-4 R	CTCTGCTCGTTTCTTTGGTGCTT3
Cor15A	qCor15A F	ATGGCGATGTCTTTCTCAGGAGCTGTT
	qCor15A R	TTTTATCCGTCACGAAATCTGAAGCTT
TEM2	qTEM2 F	GTTCTGGAAGACGGCGATTTAG
	qTEM2 R	TTTCTCGGCGTGTTGTTTAGGT
Actin2	qActin2 F	ATGCCCAGAAGTCTTGTTCC
	qActin2 R	TGCTCATACGGTCAGCGATA
